# Dietary Omega-3 Fatty Acid Deficiency and High Fructose intake in the Development of Metabolic Syndrome Brain, Metabolic Abnormalities, and Non-Alcoholic Fatty Liver Disease

**DOI:** 10.3390/nu5082901

**Published:** 2013-07-26

**Authors:** Artemis P. Simopoulos

**Affiliations:** The Center for Genetics, Nutrition and Health, 2001 S Street, NW, Suite 530, Washington, DC 20009, USA; E-Mail: cgnh@bellatlantic.net; Tel.: +1-202-462-5062; Fax: +1-202-462-5241.

**Keywords:** metabolic syndrome and insulin resistance, dietary omega-3 fatty acid deficiency, high fructose intake, non-alcoholic fatty liver disease, brain insulin resistance, hippocampus, learning, memory, appetite, inflammation, endocannabinoids, neurodegenerative diseases

## Abstract

Western diets are characterized by both dietary omega-3 fatty acid deficiency and increased fructose intake. The latter found in high amounts in added sugars such as sucrose and high fructose corn syrup (HFCS). Both a low intake of omega-3 fatty acids or a high fructose intake contribute to metabolic syndrome, liver steatosis or non-alcoholic fatty liver disease (NAFLD), promote brain insulin resistance, and increase the vulnerability to cognitive dysfunction. Insulin resistance is the core perturbation of metabolic syndrome. Multiple cognitive domains are affected by metabolic syndrome in adults and in obese adolescents, with volume losses in the hippocampus and frontal lobe, affecting executive function. Fish oil supplementation maintains proper insulin signaling in the brain, ameliorates NAFLD and decreases the risk to metabolic syndrome suggesting that adequate levels of omega-3 fatty acids in the diet can cope with the metabolic challenges imposed by high fructose intake in Western diets which is of major public health importance. This review presents the current status of the mechanisms involved in the development of the metabolic syndrome, brain insulin resistance, and NAFLD a most promising area of research in Nutrition for the prevention of these conditions, chronic diseases, and improvement of Public Health.

## 1. Introduction

Over the past century, major changes have taken place in the food composition of Western diets, in terms of essential fatty acids and sugar intake, particularly fructose [1,2,3,4,5,6]. Today’s Western diets are characterized by increases in total fat, especially in saturated fat and omega-6 fatty acids and decreases in omega-3 fatty acids in comparison to the fatty acid intake during evolution for which our genes were programmed to respond [1,3,6,7]. Omega-3 deficiency contributes to insulin resistance and the metabolic syndrome [1,8,9] brain metabolic abnormalities [10,11,12,13], liver steatosis or non-alcoholic fatty liver disease (NAFLD) [14]. An increase in fructose intake contributes to similar metabolic effects. The largest increase in the intake of fructose occurred with the introduction of high fructose corn syrup (HFCS) in soft drinks and processed foods about 30 years ago [2,5,6]. These dietary changes: the omega-3 fatty acid deficiency and the excessive fructose intake occurred as a result of agribusiness, modern agriculture and food processing, and not because there was any scientific evidence that required decreasing omega-3 fatty acid intake while increasing omega-6 fatty acids and fructose intake. With the decrease in omega-3 fatty acid intake there has been an absolute and relative increase in omega-6 fatty acids leading to an increase in the omega-6/omega-3 ratio from 1–2/1 to about 16/1 in terms of both 18, 20 and 22 carbon atoms (linoleic acid (LA), arachidonic acid (AA), alpha-linolenic acid (ALA), eicosapentaenoic acid (EPA), and dochosahexaenoic acid (DHA)) and LA/ALA and AA/EPA + DHA ratios [15]. Omega-3 fatty acids have been studied extensively since 1985 and have been shown to play an important role in growth and development and in health and disease [1,3].

In epidemiological studies, animal experiments and clinical intervention studies the consumption of HFCS, or sugar sweetened beverages has been linked to the presence of unfavorable lipid levels, high triglycerides, high small dense LDL and low HDL [4], cardiovascular disease [5], type 2 diabetes [6,16,17], insulin resistance [18,19], the metabolic syndrome and liver steatosis [14]. More recently it has been shown that moderate amounts of fructose and sucrose significantly alter hepatic insulin sensitivity and lipid metabolism compared with similar amounts of glucose [20,21,22,23]. Recent studies reveal the broad effects of the metabolic syndrome on mental health disorders, cognitive function, mood changes and depression [10,11,12]. Furthermore, diabetic and obese individuals have increased vulnerability to mental health [13]. For these reasons the effects of metabolic syndrome on brain are being extensively investigated. In this paper I review the effects of the interaction of omega-3 fatty acid deficiency and high fructose intake in the development of metabolic syndrome, brain metabolic abnormalities, cognitive function, mental health, and liver steatosis or NAFLD. Fortunately, these adverse effects may be prevented or ameliorated with dietary repletion of omega-3 fatty acids.

## 2. The Metabolic Syndrome

The Metabolic Syndrome first made its appearance as “Syndrome X” described by Reaven in his Banting lecture in 1988 [24]. Reaven described “Syndrome X” as consisting of resistance to insulin-stimulated glucose uptake, glucose intolerance, hyperinsulinemia, increased very-low-density lipoprotein triglyceride, and decreased high-density lipoprotein cholesterol and hypertension. Reaven considered insulin resistance to be of primary importance in coronary artery disease, hypertension and type 2 diabetes. Since then an explosion of publications has described the relationships among insulin resistance, hyperinsulinemia, type 2 diabetes, obesity, hypertension, and coronary artery disease [25]. Insulin resistance is a metabolic state in which insulin in physiological concentrations fails to produce a normal biologic response, and is the core perturbation in the metabolic syndrome. Obesity is a classic state of insulin resistance is present in the majority of patients with impaired glucose tolerance, in patients with type 2 diabetes, and in about 25% of non-obese healthy individuals with normal glucose tolerance [24]. Although many definitions of the metabolic syndrome have been developed by various national and international organizations, they all agree on the essential components (central obesity, insulin resistance/glucose intolerance, dyslipidemia and hypertension). Estimates from the U.S. show that the prevalence of the metabolic syndrome among adults ranges between 20% and 22%, and the prevalence of NAFLD is about 20% [26,27]. More recent estimates in the U.S. show that the prevalence of the metabolic syndrome among adults ranges from 34.3% to 38.5% depending on the criteria used to define abdominal obesity [28].

Metabolic syndrome as a risk factor for neurological disorders has been a focus of research since the observations that at the molecular level metabolic syndrome is accompanied by dysregulation in the expression of cytokines and chemokines and alterations in the levels of leptin [10]. Such changes modulate immune response and inflammation that lead to alterations in the hypothalamic “bodyweight/appetite/satiety set point” [10]. The involvement of the brain in the pathogenesis of metabolic syndrome is associated with neurochemical changes in stroke, depression and Alzheimer’s disease [13]. Furthermore, the metabolic syndrome has been associated with cognitive function and metabolic brain abnormalities [11]. In a literature search on metabolic syndome brain and cognition, Yates *et al.* [11] included studies investigating individual components of metabolic syndrome. Multiple cognitive domains were affected by metabolic syndrome in adults. In adolescents the majority of findings were in executive functioning [11]. Brain literature in adults implicated metabolic syndrome in ischemic stroke, white matter alterations, and altered brain metabolism. In adolescents, individual metabolic syndrome factors were associated with volume losses in the hippocampus and frontal lobes [11]. Potential factors include impaired vascular reactivity, neuroinflammation, oxidative stress, and abnormal lipid metabolism [10,11]. The deficiency of omega-3 fatty acids, impacts negatively on cognitive performance and brain structure, as a result of insulin resistance-associated cerebrovascular reactivity [10,11,12].

Insulin receptors are expressed in the brain and the physiological roles for insulin in the CNS are being delineated. Epidemiological evidence suggests an increased risk of developing dementia, including Alzheimer’s disease, in persons with obesity, type 2 diabetes, but also in those with poor insulin sensitivity without diabetes, suggesting a mechanistic link between adiposity, insulin sensitivity and dementia [12]. In their review, Williamson *et al.* express their concerns on the difficulties in quantifying insulin sensitivity in the brain and emphasize the need to develop technology for this purpose, so that insulin sensitivity’s role in the “new age” dementia that is related to today’s diet and lifestyle is better defined [12].

De La Monte [13] in his recent review on the role of peripheral insulin resistance in cognitive impairment and Alzheimer’s disease concludes that systemic insulin resistance diseases linked to obesity, type 2 diabetes and non-alcoholic steatohepatitis promote neurodegeneration. Insulin-resistance dysregulates lipid metabolism which promotes ceramide accumulation with attendant inflammation and endoplasmic reticulum stress. De La Monte states that “toxic ceramides generated in extra-CNS tissues, e.g., liver, get released into peripheral blood, and subsequently transit across the blood brain barrier into the brain where they induce brain insulin resistance, inflammation and cell death”.

## 3. Dietary Omega-3 Fatty Acid Deficiency

**Figure 1:**
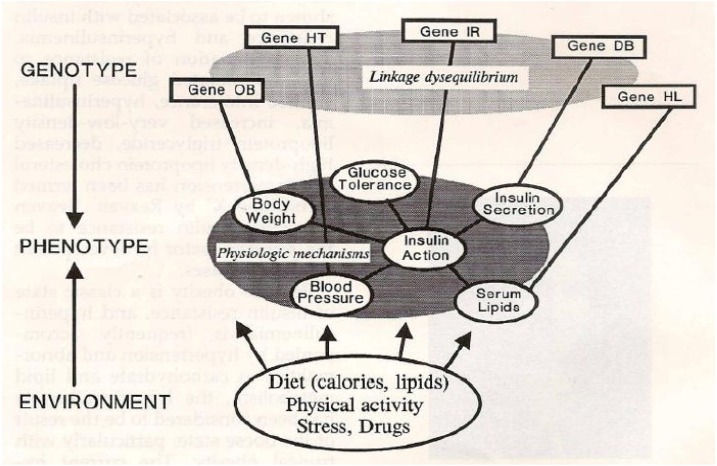
Network of interrelated functions, connected by physiological mechanisms, controlled by heredity and influenced by environmental factors [29]. (Reproduced with permission).

**Figure 2:**
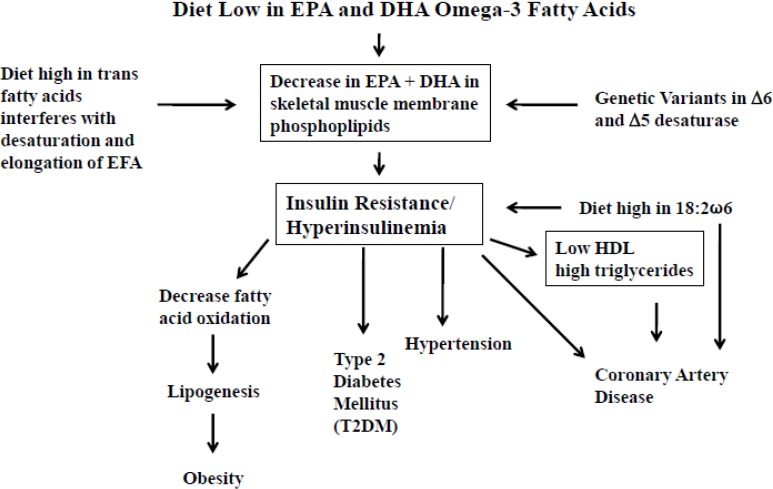
The effects of dietary eicosapentaenoic acid (EPA) + dochosahexaenoic acid (DHA) on the composition of the EPA + DHA in skeletal muscle membrane phospholipids and their relationship to insulin resistance/hyperinsulinemia and chronic diseases (obesity, T2DM, hypertension, coronary artery disease). (Modified from Reference [31]).

In 1991, Storlien *et al.* studied the influence of dietary fat composition on development of insulin resistance in rats [8]. They showed that impaired insulin action in skeletal muscle is related to triglyceride accumulation, suggesting intracellular glucose-fatty acid cycle involvement, and that long-chain omega-3 fatty acids in phospholipid of skeletal muscle may be important for efficient insulin action. In rats made insulin resistant with a high fat diet, the resistance could be prevented by the inclusion of omega-3 fatty acids in the diet, but only under circumstances in which the EPA and DHA became incorporated in the phospholipid component of the muscle cells. Subsequently, Borkman *et al.* [9] performed studies in human subjects on the relationship between insulin sensitivity and the fatty acid composition of skeletal muscle membrane phospholipids, and showed that decreased insulin sensitivity is associated with decreased concentrations of long-chain polyunsaturated fatty acids (PUFA) in skeletal-muscle phospholipids, raising the possibility, that changes in the fatty acid composition of muscle cell membrane modulates the action of insulin in human subjects as was shown earlier in rats [8]. Skeletal muscle is the principle site of insulin mediated glucose disposal and the fatty acid composition of membranes influences the action of insulin within the skeletal muscle, whereas the fasting serum insulin concentration was positively correlated with the percentages of LA indicating that high or increased LA is associated with decreased insulin sensitivity [9]. The results of this study, in conjunction with the studies in cell systems and animals, suggest that variations in insulin sensitivity are related to differences in the membrane content of long-chain PUFA within skeletal muscle phospholipids. Therefore, abnormalities in the fatty-acid composition of membranes may be involved in the pathogenesis of a cluster of disorders linked to insulin resistance and hyperinsulinemia, including obesity, hypertension, type 2 diabetes mellitus, and coronary artery disease, suggesting that diet may influence their development [1,3]. Furthermore, these conditions are known to have genetic determinants for hypertension, type 2 diabetes, some forms of hyperlipidemia, obesity, and insulin resistance (Figure 1) [29] and have a common abnormality in smooth muscle response and insulin resistance [30,31]. Figure 2 is a hypothetical scheme of how a decrease in EPA and DHA in muscle cell membrane phospholipids from (1) a decrease in dietary intake per se, (2) increased dietary intake of trans fatty acids, (3) increased intake of LA, or (4) genetic variants in delta-6 and delta-5 desaturases may lead to a decrease in EPA and DHA muscle cell membrane phospholipids [31]. Such a decrease contributes to insulin resistance and hyperinsulinemia, with the subsequent development of obesity, hypertension, type 2 diabetes, and coronary artery disease (including asymptomatic atherosclerosis and microvascular angina) [31].

A decrease in brain and retina DHA, resulting from nutritional deficiency or aging is associated with reduced cognitive ability, increased emotional behavior and decreased visual capacity [32]. Epidemiological and clinical data link omega-3 fatty acid deficiency with mood disorders [33].

The endocannabinoid system includes the two endogenous ligands 2-arachidonoylglycerol (2-AG) and *N*-arachidonoylethanolamine (anandamide or AEA) and two cannabinoid receptors (CB_1_ and CB_2_). Central CB_1_ receptor activation by increased endocannabinoid levels or exogenous agonists induces hyperphagia along with increased lipogenesis and peripheral adiposity [34,35]. CB_1_ receptors are predominantly expressed in the brain, but also in the peripheral tissues of the gastrointestinal tract, adrenal glands, liver, adipose tissue, and skeletal muscles [36]. In the liver, CB_1_ activation increases de novo lipogenesis through stimulation of cytosolic fatty acid synthase activity leading to fatty liver and obesity [35]. Pharmacological blockade of the CB_1_ receptor is effective in treating obesity and related metabolic derangements. However serious psychiatric side effects, including depression and suicidal tendencies [37], caused marketplace withdrawal of Rimonabant, a selective CB_1_ antagonist [38]. Endocannabinoids are endogenous lipid mediators made from essential fatty acids available only from dietary sources. The two best characterized endocannabinoids, 2-AG and AEA, are both metabolic derivatives of a single fatty acid precursor AA in phospholipids. Endocannabinoids are formed enzymatically on demand from the pool of AA plasma phospholipids formed from LA or ingested as AA. In a study on mice Alvheim *et al.* [39] showed that dietary linoleic acid increased tissue AA which led to increased 2-AG + 1-AG and AEA resulting in the development of diet induced obesity by increasing food intake, feed efficiency and adiposity in mice. Reducing LA in the diet and adding EPA + DHA reduced the AA phospholipid pool, normalized endocannabinoid tone and reversed the obesity. Obesity is associated with neurological morbidity, including major depression [40], but the underlying pathophysiological mechanisms remain poorly understood. Lafourcade *et al.* [41] used a specific diet to mimic lifelong omega-6/omega-3 imbalance of essential fatty acids in mice and showed that reducing omega-3 levels markedly diminished the synaptic and behavioral functions of the cannabinoid CB_1_R. Omega-3 deficiency profoundly affected emotional behaviors in the rodents and support the clinical and epidemiological associations between omega-6/omega-3 imbalance and mood disorders [33,41]. The authors concluded “by linking diet to altered synaptic functions of the CB_1_R in relevant brain areas, our data provide the first synaptic substrate for the impairment of emotional behavior, including depression, associated with the low levels of *n*-3 PUFAs that are frequently observed in Western diets” [41].

## 4. High Fructose Intake

There has been a shift in the types of nutrients consumed in the American diet. The consumption of fructose has increased mostly because of an increased consumption of soft drinks and many other beverages that are high in fructose and because of the consumption of foods such as breakfast cereals, baked goods, condiments, and prepared desserts all sweetened with sucrose and high-fructose corn syrup (HFCS) [2]. HFCS is produced by the enzymatic isomerization of dextrose to fructose [2]. The commercial use of HFCS began to increase in the 1970s and by 1985, HFCS accounted for ~35% of the total amount of sweeteners by dry weight in the food supply [2]. Although HFCS can contain up to 90% fructose, most of the HFCS used in beverages contains ~55% fructose [2,4]. Young males, 15–18 years of age, reported the highest intake of fructose intakes with the 90th percentile intake from all sources of about 100 grams per day [2]. The use of crystalline fructose has been expanded to the general food supply. The use of glucose syrup also increased whereas the contribution of other sweeteners, supplied as honey, molasses and maple syrup remained constant at 1% [2].

**Figure 3:**
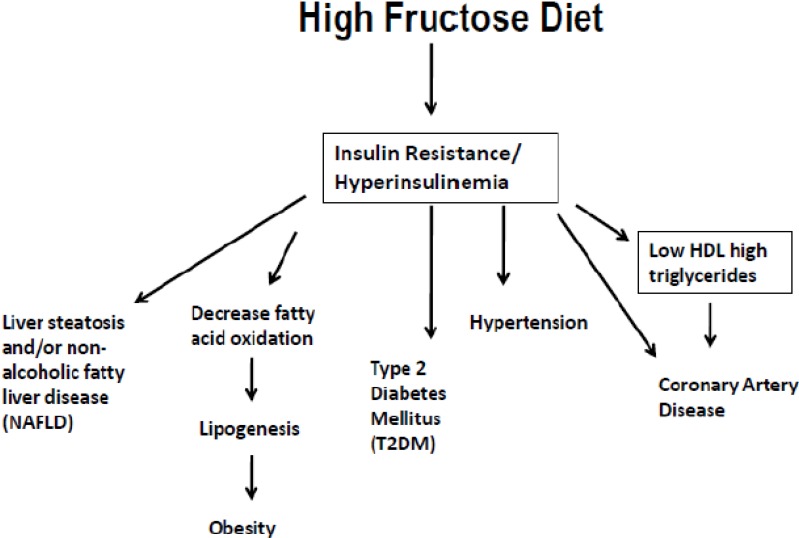
The effects of dietary Fructose and its relationship to insulin resistance/hyperinsulinemia and chronic diseases (liver steatosis, obesity, T2DM, hypertension, coronary artery disease).

Over the past 35 years concerns have been expressed about the adverse effects of excessive consumption of sugar (Table 1) (Figure 3). From 2001 to 2004 the usual intake of added sugar in the American diet was 22.2 teaspoons per day, which is 335 calories per day [2].

In 2003 the WHO report on Diet and Chronic Diseases recommended that added sugar should not constitute more than 10% of energy intake [42]. Dietary sugars contribute to the development of obesity [2]. A large number of calories are obtained from beverages, processed food, and deserts made with sucrose or high fructose corn syrup, which are absorbed as a mixture of glucose and fructose. Apart from weight gain from excess sweet calories there are a number of metabolic consequences harmful to health. The best documented adverse effects known for decades is the dyslipidemia that develops with the ingestion of large amounts of sugar even when substituted calorie for calorie for fat [43]. An important mechanism is the de-novo lipogenesis (DNL) synthesis of the saturated fat palmitate from glucose, fructose or both. There is a marked increase in DNL after excess carbohydrate calories [44] leading to high triglycerides, small dense LDL and low HDL that accelerate atherosclerosis. The recent study by Sevastianova *et al.* [45] expands the lipogenic effects of dietary sugar beyond dyslipidemia to include fatty liver. A 2% increase in body weight and similar increases in subcutaneous and visceral adipose tissue were measured by proton magnetic resonance spectroscopy. The results showed for the first time a link between excess dietary sugar and the accumulation of liver fat by DNL a pathway uniquely stimulated by dietary sugar. There was also an increase in fasting insulin level.

In the U.S., hepatic steatosis affects a large fraction of obese adults and children, may progress to non-alcoholic steatohepatitis, cirrhosis, and liver failure. The implication that a persistent excess of calories as dietary sugar can cause or exacerbate fatty liver by DNL lends additional support to public health recommendations to limit intake of sugars [2,42]. The increased liver fat resulting from increased DNL and the imbalance between triglyceride synthesis and secretion may increase oxidative stress, inflammation and insulin resistance (Figure 3) [4]. In 2009, the American Heart Association Nutrition Committee recommended that women and men consume no more than 100 and 150 kcal of added sugar per day, respectively [2]. In 2011, Stanhope *et al.* [46] compared the effects of consuming glucose, fructose, or high-fructose corn syrup (HFCS) at 25% of energy requirements on risk factors for cardiovascular disease, and concluded that “consumption of HFCS sweetened beverages for 2 weeks at 25% of energy increased risk factors for cardiovascular disease comparably with fructose and more than glucose in young adults” [46]. This well executed study contradicts the conclusions from recent reviews that sugar intakes as high as 25%–50% of energy, have no adverse longtime effects with respect to components of the metabolic syndrome [47] and that fructose consumption up to 140 g/day does not result in biologically relevant increase of fasting or postprandial triglycerides in healthy, normal-weight [48] or overweight or obese [49] humans. These reviews [47,48,49] unfortunately influenced the decision of the Committee of the 2010 dietary guidelines for Americans in which maximal intake levels of 25% or less of total energy from added sugars is suggested instead of the 10% level of the recommendation of the WHO/FAO report [42]. Stanhope *et al.* [50] clearly showed the twenty-four hour triglyceride area under the curve increased compared with baseline during fructose consumption and HFCS but not with glucose. Fasting LDL and apoB concentrations were increased during consumption of fructose and HFCS but not with glucose indicating that the adverse effects associated with chronic consumption of sugar-sweetened beverages results from the specific effects of fructose [39] rather than from circulating glucose and insulin excursions [22,51,52,53,54]. In 2009, Stanhope *et al.* had shown that consumption of fructose-sweetened beverages at 25% of energy increased visceral adipose tissue and de novo lipogenesis, produced dyslipidemia, and decreased glucose tolerance/insulin sensitivity in older, overweight/obese men and women, whereas glucose-sweetened beverages did not [4]. Similarly Silbernagel *et al.* [55] reported that consumption of fructose or glucose (150 g/day) for 4 weeks lowered insulin sensitivity and increased HOMA-IR in subjects of similar age (31 ± 9 years) and BMI = (25.9 ± 22).

In terms of their composition commonly ingested sugars, sucrose and HFCS consist of 50%–55% fructose [2]. In most studies, the fructose and HFCS at 25% of energy but not glucose lead to significant increases in postprandial TG, and fasting and postprandial concentrations of LDL, apoB, and the apoB to apoAI ratio all established risk factors of CVD. At present, U.S. food labels contain information on total sugars per serving but do not distinguish between sugars that are naturally present in foods and added sugars. Thus, it is impossible for consumers to determine the amount of added sugars in foods or beverages or some drugs and cough syrups. However, a few sources of information do exist on added sugar content. The Department of Agriculture in the U.S. published a database for the added sugar content of selected foods in 2006 [56]. In addition, several voluntary food-labeling systems are in place or are being developed, some of which include criteria for limiting added sugars. These include the Smart Choices Program [57], the Hannaford’s grocery chain Guiding Stars program [58], the Overall Nutrient Quality Index [59], and the Nutrient Rich Foods Coalition nutrient density score [60].

It is ironic indeed, that originally fructose was proposed as the ideal sweetener for persons with Type 2 Diabetes because it does not raise blood sugar and is unable to stimulate insulin secretion. Fructose is now implicated in the epidemics of obesity and diabetes type 2, insulin resistance, hypertension, visceral adiposity, and the metabolic syndrome [2,4]. The effects of fructose on fasting triglycerides may be higher in women, especially post menopausal women than in men [2], in sedentary overweight people [61], in those with the metabolic syndrome [62] and in those eating low fiber diets [62].

Mechanisms by which fructose increases fasting and postprandial triglyceride levels are: increased de novo lipogenesis in the liver [4,62,63], increased hepatic triglycerides synthesis, and secretion of very-low-density lipoproteins [64]; reduced lipoprotein lipase activity at the adipocyte, which decreases the rate of peripheral triglyceride clearance [64]. A high consumption of sugar-sweetened beverages and foods is associated with evidence of increased inflammation and oxidative stress [65,66]. Others have shown that fructose may slow the basal metabolic rate in mice [67]. Fructose is the only sugar that raises uric acid concentrations [68]. Fructose, compared with glucose, is preferentially metabolized to lipid in the liver [4]. Fructose consumption induces insulin resistance, impaired glucose tolerance, hyperinsulinemia, hypertriglyceridemia, and hypertension in animal models [4] (Table 1).

**Table 1:** Summary of adverse effects of sugar and sugar-sweetened beverages (fructose).

Contributes to Metabolic Syndrome: Unfavorable lipid levels, high triglycerides, low HDL, high small dense LDL [2,4]. Increases Insulin Resistance [2,4]. Increases Obesity (Visceral Adiposity) [2,4]. Increases Type 2 Diabetes [2,4]. Leads to Fatty Liver [2,4]. Increases Cardiovascular Disease (includes Hypertension) [2,4,61,67]. May slow basal metabolic rate [67]. Increases de novo lipogenesis [43,44,62,63]. Increases hepatic triglyceride synthesis and secretion of very-low-density lipoproteins [64]. Reduces lipoprotein lipase activity at the adipocyte, which decreases the rate of peripheral triglyceride clearance [64]. Decreases glucose tolerance/insulin sensitivity [65]. Increases Inflammation [65]. Increases Oxidative Stress [66]. Fructose is the only sugar that raises uric acid concentrations [61,68]. Fructose reduces circulating insulin and leptin and attenuates postprandial suppression of ghrelin, all of which affect the satiety center of CNS (continue to eat) contributing to excess energy intake [13].

Vartanian *et al.* carried out a meta-analysis reviewing 88 cross-sectional and prospective studies evaluating the relationship between soft drink intake and nutrition on health outcomes [69]. Higher intake of soft drinks was associated with greater energy intake, higher body weight, lower intake of other nutrients and worse health indices. Further analyses from a larger trial confirmed these findings, specifically greater weight loss as sugar-sweetened beverage intake decreased [70].

## 5. Dietary Omega-3 Deficiency, High Fructose Intake, Insulin Resistance, and the Brain

The Omega-3 fatty acids, EPA and DHA have been shown to be essential for visual function and cerebral maturation of the infant and to play an important role in improving mental health, learning and memory, neurogenerative diseases, depression and schizophrenia [71]. Sucrose infusion directly into the nucleus accumbens alters dopamine and opioid neurotransmission, increasing food intake in rats [72]. Both sweet and high fat foods mobilize opioids and dopamine within the nucleus accumbens, establishing hard-wired pathways for craving in these areas [73,74]. Chronic hyperinsulinemia may also contribute to increased caloric intake by preventing dopamine clearance from the nucleus accumbens, fostering pleasure derived from food in situations in which energy states are replete, contributing to excess energy intake [75]. Teff *et al.* [76] showed that dietary fructose reduces circulating insulin and leptin and attenuates postprandial suppression of ghrelin all of which affect the satiety center in the Central Nervous System (CNS). Page *et al.* [77] studied the effects of fructose *vs*. glucose on regional cerebral blood flow in brain regions involved with appetite and reward pathways in healthy volunteers and found that the animal studies on the brain effects of fructose on appetite promotion are relevant to the humans. The major new finding is that hypothalamic brain signal generated in response to fructose ingestion was statistically different from the response following glucose ingestion [77]. Thus, after glucose consumption there is an increased sensation of fullness and satiety but not after fructose consumption suggesting that when the human brain is exposed to fructose, neuro-biological pathways involved in appetite regulation are modulated thereby promoting food intake.

Due to the central role of brain in the metabolic control of many diseases, the role of brain endoplasmic reticulum (ER) stress in metabolic disease has come into focus recently. Expanding on the previous knowledge that brain ER stress underlies neurodegenerative diseases [78] a number of studies have causally linked brain ER stress to the development of metabolic syndrome and related disorders such as overeating, obesity, leptin resistance, insulin resistance, beta-cell dysfunction and hypertension [79,80,81,82] under conditions of overnutrition [79,83] and related inflammatory insults [82]. The CNS, particularly hypothalamus, is the central regulator of energy and body weight balance [84,85,86,87,88,89,90,91,92,93,94,95,96,97,98,99].

Insulin receptors are expressed in the brain and the physiological roles of insulin in the CNS are starting to be delineated [12]. Disrupted neuronal insulin action may underlie the link between diabetes and neurodegenerative disorders [100]. Longitudinal studies have identified a higher risk of dementia or cognitive decline associated with type 2 diabetes and also in insulin resistance without diabetes [100]. Obesity has a major influence on the development of insulin resistance, and is considered the underlying cause of the current epidemic of type 2 diabetes mellitus (T2DM) [2]. At this time it is not clear what molecular lesions in liver, muscle, and adipose tissue of patients is responsible for the development of insulin resistance and ultimately T2DM, or if every tissue develops insulin resistance by the same route. The molecular pathology of insulin resistance remains controversial. It could be related to the development of a post-receptor deficit, reducing the insulin “sensing” or signaling capacity of individual cells [101]. This, in turn, results in a requirement for higher levels of insulin to stimulate glucose uptake into muscle, to reduce glucose production in liver and to correctly regulate adipose tissue. The analysis of intracellular signaling pathways in specific tissue may prove to be a means to establish the insulin sensitivity of each tissue. This is most important in the more unusual insulin target tissues such as the brain, where tissue insulin sensitivity currently cannot be assessed by HOMA or clamp techniques.

Agraval and Gomez-Pinilla [102] stimulated by the recent research revealing the broad effects of metabolic syndrome on mental health, cognitive function, and mood, via studies which showed that diabetic and obese individuals have increase vulnerability to mental health disorders, investigated the effect of DHA to counteract the effects of metabolic syndrome in the CNS in adult male Sprague-Dawley rats. Abundant consumption of fructose is an important contribution to the metabolic syndrome [103]. Studies have shown that rats fed on a high fructose diet develop hepatic oxidative damage and altered lipid metabolism as a result of the burden of fructose metabolism [104]. Agraval and Gomez-Pinilla [102] randomly assigned six animals on an omega-3 fatty acid diet (0.5% flaxseed oil (ALA) and 1.2% DHA) or omega-3 deficient diet with or without fructose solution 15% as drinking water for 6 weeks. The total fat content in both diets was 10 g per 100 g diet. There were no differences among the groups on body weight, calorie intake, and food and water consumption. The deficiency of omega-3 fatty acids resulted in a significant increase in latency time with the Barnes maze test, indicating memory impairment, which was further enhanced by fructose intake. The effects of fructose on memory in the omega-3 deficient rats was ameliorated by the omega-3 diet indicating that dietary omega-3 fatty acid deficiency influences the vulnerability for fructose induced changes. In the omega-3 deficient animal there was an increase in triglycerides that was further increased by fructose intake. Fructose also increased the glucose and insulin levels in the omega-3 deficient animal. The presence of omega-3 fatty acids in the diet reduced the fructose increase in insulin and triglyceride levels. There was a correlation between serum triglycerides and insulin resistance index and between triglycerides and fructose induced memory deficits suggesting that memory performance may rely on levels of insulin resistance index. The deficient omega-3 fatty acid diet and fructose led to a decrease in pTyr1R levels in the hippocampus which was reversed by the omega-3 diet. There was a negative correlation between insulin resistance levels and pTyr1R suggesting that the increased insulin resistance in the body may disrupt insulin receptor signaling in the brain. The Akt phosphorylation was found to be decreased with omega-3 fatty acid deficiency which was exacerbated by fructose intake but was alleviated by Akt phosphorylation in the presence of the omega-3 fatty acid diet. The omega-3 fatty acid deficient diet led to decreased phosphorylation of LKB1 whereas the omega-3 fatty acid diet increased the phosphorlyation of LKB1. There was also a positive correlation between phosphorylated LKB1 and DHA and a negative correlation with AA pointing to a concomitant alteration of LKB1 after diet treatment and the altered lipid composition in brain (Table 2).

**Table 2:** Summary of adverse effects of omega-3 deficient diets and high fructose, corrected by the omega-3 diet [102].

Factor	Omega-3 Deficient Diet	Fructose	Omega-3 Diet
Increase in latency time in the Barnes Maze Test indicating memory impairment	↑	Potentiates this effect	Ameliorates
Triglycerides	↑	Potentiates this effect	Ameliorates
Insulin Resistance	↑	Potentiates this effect	Corrects
pTyrIR levels in Hippocampus	↓	Decreases this effect	Reversed
Akt phosphorylation	↓	Exacerbates this effect	Alleviates
Phosphorylation in KLβ1	↓	____	Increases
Phosphorylation of CREB	↓	Exacerbates this effect	Counterregulated the fructose induced alteration in synaptic plasticity via CREB Synapsin 1 Synaptophysin
Hippocampus and Frontal lobe volume	↓	____	____
Sir2	↓	↓	Normalizes
Liver Steatosis	↑	↑	Reverses

Omega-3 deficiency showed decreased phosphorylation of CREB which was further exacerbated by fructose treatment. The presence of omega-3 fatty acids counter regulated the fructose induced alteration in synaptic plasticity via CREB. Similar results were obtained with Synapsin I and Synaptophysin. Omega-3 fatty acid deficient diet makes the brain more vulnerable to fructose induced free radical attack. Clinical studies indicate that a balanced ratio of omega-6/omega-3 is important to maintain health and normal development [1]. The data show that omega-3 deficiency with or without fructose promoted a decrease in the phosphorylation of LKB1, indicating that activation of LKB1 may rely on levels of omega-3 fatty acid in the brain. The changes in LKB1 phosphorylation varied in direct proportion to DHA level and inverse proportion to AA level, which suggests that a decline in the ratio of AA/DHA contributes to energy homeostasis (Table 2).

## 6. Dietary Omega-3 Deficiency and High Fructose Intake in the Development of Non-Alcoholic Fatty Liver Disease (NAFLD)

As shown earlier in this paper high fructose intake leads to obesity, insulin resistance and in comparison to glucose, is preferentially metabolized to lipids in the liver increasing triglyceride synthesis while decreasing their secretion leading to NAFLD (Table 1). Numerous *in-vitro* and *in-vivo* studies have demonstrated that omega-3 fatty acids are able to coordinate both the upregulation of lipid oxidation by binding and activating peroxisome proliferator activated receptor α (PPARα) [105,106], and the downregulation of lipid synthesis-suppressing lipogenesis by inhibiting sterol regulatory element binding protein-1c (SREBP-lc) gene expression and for activation by proteolysis [106,107,108]. Several clinical studies have reported the beneficial effects of EPA and DHA supplementation on triglyceridemia [109] blood pressure [110] inflammation [111] and insulin sensitivity [30,31]. A lower intake of omega-3 fatty acids was suggested to be associated with NAFLD [112,113]. Experiments in rats and mice that were omega-3 deficient for two generations displayed several features of the metabolic syndrome including hepatic steatosis [114,115]. Pachikian *et al.* [116] investigated in mice the effect of omega-3 depletion for 3 months on hepatic lipid composition and metabolism using molecular integrative and physiological approaches *in-vitro* and *in-vivo*. They observed a stimulation of the hepatic lipogenic pathway most likely induced by the increased expression and activity of SREBP-1c. Specifically this study showed (1) decreased omega-3 fatty acids in the phospholipid fractions and changes (increases) in hepatic endocannabinoid content and AA; (2) omega-3 fatty acid depletion decreased fatty acid oxidation and promoted hepatic lipid synthesis and storage; (3) microarray analysis confirmed a metabolic shift in favor of fatty acid and cholesterol synthesis at the expense of fatty acid oxidation in the livers of omega-3 fatty acid depleted mice; (4) SREBP-1c is involved (higher expression, activation) in the metabolic alterations occurring in the livers of omega-3 fatty acid depleted mice; (5) mice depleted of omega-3 fatty acids displayed hepatic insulin resistance as shown by the higher hepatic glucose production upon insulin stimulation when compared with control mice (by euglycemic hyperinsulinemic clamp); (6) omega-3 fatty acid depletion did not induce hepatic endoplasmic reticulum (ER) stress; (7) increased liver X receptor (LXR) activity occurred in the livers of omega-3 fatty acid depleted mice. Insulin is considered to be the classical driver of SREBP-1c activation which largely explains carbohydrate induced lipogenesis [117]. Because there were no changes in insulin levels, omega-3 fatty acid depletion promoted insulin resistance by an insulin independent pathway. This study demonstrated that the metabolic characteristics in this model of omega-3 fatty acid depletion are opposite to the ones occurring with omega-3 fatty acid supplementation [118]. The consumption of a diet containing low levels of omega-3 fatty acids for 3 months was sufficient to induce hepatic omega-3 fatty acid depletion in phospholipids, steatosis and insulin resistance. Decreased fatty acid oxidation and increased triglyceride and cholesterol synthesis both contributed to lipid accumulation. Because the activation of SREBP-1c related pathways occurred in a hepatic insulin resistant state and independently of ER stress, it is consistent with increased liver X receptor (LXR) activity, and a higher endocannabinoid ligand level (2-AG). The SREBP-1c results in human biopsies of NAFLD patients are characterized by an increased expression of SREBP-1c and decreased expression of PPARα in patients with omega-3 depletion [119]. In an elegant study, Gonzalez-Periz *et al.* [14] investigated the effect of omega-3 fatty acid supplementation in the *ob/ob* mice, an obesity model of insulin resistance and fatty liver disease. They showed that dietary intake of omega-3 fatty acids had insulin-sensitizing actions in adipose tissue and lower and improved insulin tolerance in obese-mice. Omega-3 fatty acids upregulated the genes involved in insulin sensitivity (PPARα), glucose transport (GLUT-2/GLUT-4) and insulin receptor signaling (IRS-1/IRS-2). Furthermore omega-3 fatty acids increased adiponectin, and induced AMPK phosphorylation, a fuel-sensing enzyme and a gatekeeper of the energy balance. At the same time hepatic steatosis was alleviated by omega-3 fatty acids. Lipidomic analysis showed that omega-3 fatty acids inhibited the formation of omega-6 derived eicosanoids, while induced the formation of omega-3 derived resolvins and protectins from EPA and DHA respectively. Resolvin E1 and protectin D1 mimicked the insulin-sensitizing and antisteatotic effects of omega-3 fatty acids, and induced adiponectin expression to a similar extent as that of rosiglitazone- an antidiabetic drug. This study clearly showed the beneficial effects of omega-3 fatty acids and their lipid autacoids (resolvins + protectins) in preventing obesity induced insulin resistance and hepatic steatosis.

## 7. Conclusions, Health Implications and Recommendations

Long term consumption of western diet which is high in saturated fat, omega-6 fatty acids and sugar especially fructose, while low or deficient in omega-3 fatty acids contributes to the development of metabolic syndrome including metabolic syndrome of the brain and NAFLD. The metabolic syndrome is a complex entity consisting of a constellation of metabolic risk factors including central (or vascular) obesity, insulin resistance/impaired glucose tolerance, dyslipidemia (hypertriglyceridemia and low HDL-C) and hypertension associated with an atherogenic, procoagulant and inflammatory state. The concept of the metabolic syndrome had been mainly associated with the “body”. Recent studies, however, have provided data that expand the traditional concept to include the effects of metabolic syndrome in the brain. New evidence supports the harmful impact of the metabolic syndrome on the brain, impacting synaptic plasticity and cognitive function. Deficiency of dietary omega-3 fatty acids increases vulnerability to impaired cognitive functions, and intake of high fructose diet exacerbates this condition. In terms of public health, it is encouraging that the unhealthy effects of sugars and especially fructose leading to insulin resistance, metabolic syndrome, brain metabolic abnormalities, and NAFLD can be counteracted or ameliorated by omega-3 fatty acids. Thus, the right combination of foods is crucial for brain health as well as for overall health. In animal experiments, the omega-3 supplementation was indeed essential for normalizing the phosphorylation of CREB and Synapsin I and Synaptophysin even in the presence of fructose, suggesting that omega-3 fatty acids can restore the cognitive function under challenging conditions by normalizing the action of insulin resistance on synaptic plasticity.

Epidemiological studies, clinical trials, animal experiments and cell studies indicate the need for change by the Agricultural food producers and processing industries. We must avoid omega-3 fatty acid deficient diets and excess fructose intake if we are going to decrease the risk of obesity, insulin resistance, diabetes, atherosclerosis, hypertension, depression and deficits in learning, executive function, memory and neurodegenerative diseases. Inflammation is an important consequence of the metabolic syndrome [120]. An inflammatory state in brain regulatory centers, such as hypothalamus, disrupts its metabolic function that leads to neural and neuroendocrine regulation of a number of physiological processes such as energy balance, glucose metabolism, insulin resistance, cardiovascular disease, and obesity. Although current understanding of the central inflammatory mechanisms of metabolic syndrome and related diseases are in an early stage, this area of research is very promising [121]. Omega-3 fatty acid deficient and high fructose diets are the result of agribusiness and modern agriculture that led to the increased production of omega-6 rich vegetable oils, an absolute and relative deficiency of omega-3 fatty acids and to the expansion of processing of food ingredients that led to increases in high fructose intake for the first time in human’s evolutionary history.

Recent studies with human volunteers indicate that it is essential to begin modifications of the current Western diet as follows:

1. Increase the intake of omega-3 fatty acids ALA, EPA, and DHA, and decrease LA and AA [122]. This can be accomplished by changing the oils in the Western diet and substituting the high omega-6 oils (corn oil, sunflower, safflower, soybean) with low omega-6 fatty acids and oils high in omega-3’s in order to balance the omega-6/omega-3 ratio such as (1) Olive oil which is low in omega-6 fatty acids (LA 6%–12%), (2) Canola oil which has a ratio of omega-6/omega-3 of 2:1. (3) Chia and Perilla oil which contain 55%–60% omega-3 fatty acids. Industry has recognized the need to change the oils by decreasing the high omega-6 content through genetic manipulation, *i.e.*, by developing high monounsaturated sunflower oil. Furthermore through genetic engineering of soybeans, by increasing Stearidonic acid, industry has increased the omega-3 content of soybean oil which has been shown in animal and human studies to be more effective than its precursor, α-linolenic acid, to be metabolized to EPA, thus enriching membrane phospholipids with EPA. Hence, stearidonic acid can serve as a “pro-eicosapentaenoic acid” [123,124]. It is necessary that food labels state the levels of omega-6 and omega-3 fatty acids separately instead of simply reporting them as PUFA. Furthermore studies and all journal publications should distinguish the concentration of omega-6 and omega-3 fatty acids instead of simply PUFA. The omega-6 and omega-3 fatty acids are physiologically and metabolically distinct and have opposing properties. Therefore, their balance is important for health.
2. Decrease the amount of added sugar to less than 10% of energy intake, and remove high fructose corn syrup from sweetened beverages, snacks, cookies and other forms of processed foods. Again, industry has recognized the detrimental effects to health, resulting from high fructose intake and is searching for natural ingredients low in sugars (and fructose) to substitute for HFCS. Furthermore, the levels of glucose and fructose should be stated in all food labels [125,126,127,128]. At present, U.S. food labels contain information on total sugars per serving but do not distinguish between sugars that are naturally present in foods and added sugars. Thus, it is impossible for consumers to determine the amount of added sugars in foods or beverages, or some drugs and cough syrups.
